# Chronic social stress increases nitric oxide-dependent vasorelaxation in normotensive rats

**DOI:** 10.2478/v10102-010-0049-4

**Published:** 2010-12

**Authors:** Angelika Puzserova, Iveta Bernatova

**Affiliations:** Institute of Normal and Pathological Physiology, Centre of Excellence for Cardiovascular Research, Slovak Academy of Sciences, Bratislava, Slovak Republic

**Keywords:** ascorbic acid, crowding, endothelium, social stress, vasoconstriction

## Abstract

The aim of this study was to examine oxidative load and endothelium-dependent vasorelaxation in the serotonin pre-constricted femoral artery (FA) of Wistar-Kyoto (WKY) rats exposed to chronic social stress produced by crowding in the presence or absence of ascorbic acid (AsA) in working solution. Adult male rats were randomly divided into control (living space: 480 cm^2^/rat) or stressed (living space: 200 cm^2^/rat) groups for 8 weeks. Blood pressure and heart rate, determined using tail-cuff plethysmography, were not influenced by stress vs. control. Conjugated dienes (CD) and concentrations of thiobarbituric acid-reactive substances (TBARS) were measured in the left ventricle and liver (for assessment of oxidative load) and were found unchanged by chronic crowding. The nitric oxide (NO)-dependent component of endothelium-dependent relaxation was investigated in the FA using a wire myograph. In both the presence and absence of AsA, acetylcholine-induced relaxation of the FA of stressed rats significantly exceeded that of the controls, which was associated with an increase of the NO-dependent component. In conclusion, the data showed that chronic crowding did not produce oxidative stress in the organs investigated and indicate that elevation of NO production during chronic stress is an important way of adaptation, which may prevent normotensive rats from the development of stress-induced hypertension.

## Introduction

Chronic stress and stressful life events are generally considered to be risk factors of several diseases, including hypertension (Kopp & Rethelyi, 2004). Although the mechanisms involved in stress-related hypertension are not well-defined, there are studies suggesting the involvement of peripheral vascular changes, which may significantly contribute to the pathogenesis of hypertension. On the other hand, several investigators have proposed that reactive oxygen species may participate in the development of endothelial dysfunction, which may contribute to elevated vascular resistance in stress conditions.

Despite the fact that in socially organized mammals including man a significant portion of stress arises from their interaction with the social environment, experimental stress models are mostly acute or repeated and relatively short-termed (Kopp & Rethelyi, 2004), not allowing the development of vascular adaptation mechanisms. We used chronic crowding, a relatively mild stressor, which however affects considerably the hypothalamic-pituitary-adrenal (HPA) axis, the sympathoadrenal system (Bugajski, 1999; Djordjevic *et al*., 2003; Dronjak *et al*., 2004), as well as the vascular system (Puzserova *et al*., 2006; Bernatova *et al*., 2007a, 2007c).

A number of vascular diseases, including hypertension, are characterized by endothelial dysfunction caused by alterations in the production and bioavailability of the endothelium-derived relaxing (EDRFs) and constricting factors (Torok, 2008). The functional status of the endothelium is usually tested by the acetylcholine test in pre-constricted isolated arteries. At least three different vasodilating agents are released by the endothelium after exposure to acetylcholine (ACh) – nitric oxide (NO), prostacyclin (PGI_2_), and endothelium-derived hyperpolarizing factor(s) (EDHFs) (Stankevicius *et al*., 2003). NO is synthesized from L-arginine (L-Arg) by at least three isoforms of nitric oxide synthase (NOS) (Cacanyiova *et al*., 2009). Recently we provided evidence that chronic crowding activated NOS in the aorta and improved ACh-induced relaxation of Wistar-Kyoto rats (Puzserova *et al*., 2006; Bernatova *et al*., 2007c). Thus the L-Arg/NO system supposedly protects normotensive rats from stress-induced hypertension. On the other hand, elevated NO production need not be always associated with better NO bioavailability because increased concentration of reactive oxygen species, documented in acute and chronic stress (Sivonova *et al*., 2004; Zafir & Banu, 2007, 2009; Kwiecien *et al*., 2008), can inactivate NO and thus result in endothelial dysfunction (Torok, 2008; Bernatova *et al*., 2009).

In our previous experiments, stress-related vascular reactivity was measured in the presence of ascorbic acid (AsA, 1100µmol/l) in working solution (Puzserova *et al*., 2006; Bernatova *et al*., 2007a, 2007c) to prevent oxidation of catecholamines (Hansen & Nedergaard, 1999; Bernatova *et al*., 2009). However, ascorbic acid possesses antioxidant properties and it might artificially reverse endothelial dysfunction by improvement of oxidative status resulting in better NO bioavailability (May, 2000; Bernatova *et al*., 2009). On the other hand, it has been assumed that high concentrations of AsA could have pro-oxidative effects (Durackova, 2010). Therefore the aim of this study was to determine the oxidative load in stressed normotensive rats and to investigate whether improvement of NO-dependent vasorelaxation in crowded rats is present also in the absence of ascorbic acid in working solution.

## Methods

### Animals

All rats used in the present study were born in our certified animal facility in order to maintain the same environmental background of all animals. The rats were housed in an air-conditioned room at constant temperature (22–24°C) and humidity (45–60%) at a 12:12-h light/dark cycle (06:00–18:00 h lights on) and maintained on a standard pellet diet and tap water *ad libitum*. All procedures used were in accordance with the institutional guidelines and they were approved by the State Veterinary and Food Administration of the Slovak Republic.

### Experimental design and stress model

At the beginning of the experiment, 12-week-old Wistar-Kyoto (WKY) rats were randomly divided into a control and astressed group. Controls were kept in groups of 4 rats per cage (35/55/20 cm). Rats exposed to crowding stress were kept in groups of 5 rats per cage (25/40/15 cm) for eight weeks, with their living space reduced from 480 to 200 cm^2^/rat (Bugajski, 1999; Bernatova & Csizmadiova, 2006).

After 8 weeks of experiment, the rats were killed by decapitation after a brief CO_2_ anesthesia between 7:30 and 9:30 a.m. Wet mass of the left heart ventricle (LVM) and tibial length (TL) were determined for calculation of the relative left ventricular mass (LVM/TL) to evaluate the degree of cardiac hypertrophy (Yin *et al*., 1982) independently of body weight.

### Blood pressure and heart rate

Systolic blood pressure (BP) and heart rate (HR) were determined non-invasively in conscious rats by tail-cuff plethysmography (using the Statham Pressure Transducer P23XL, Hugo Sachs, Germany) before experiment (basal) and after the 1^st^, 3^rd^, 6^th^ and 8^th^ week of experiment. One week before experimentation, the rats were handled and accustomed to the tail-cuff procedure of blood pressure recording in three independent sessions, in order to minimize non-specific stress. Blood pressure and heart rate were determined between 9:00–12:00 h and were calculated as the average value of 5–6 measurements. The study presents the values of BP and HR from the beginning (12-week-old rats) and the end of the experiment (20-week-old rats).

### *In vitro* assessment of vascular reactivity by wire myograph

Femoral arteries from the left hind limb were carefully dissected, immediately immersed in modified cold physiological salt solution (PSS) and cleaned of adipose or connective tissue. Arteries were then cut into segments (1.33 ± 0.06 mm long) and mounted as ring-shaped preparations in the Mulvany–Halpern style small vessel wire myograph (Mulvany & Halpern, 1977) chamber (Dual Wire Myograph System 410A, DMT A/S, Aarhus, Denmark) to determine the vascular reactivity during isometric conditions. The procedures for investigation of small vessels using wire myograph and apparatus have been described in detail elsewhere (Mulvany & Halpern, 1977). Briefly, two 40 µm stainless steel wires were passed through the lumen of the vessel and mounted in the jaws of the wire myograph. After 30-min equilibration in oxygenated (5% CO_2_, 95% O_2_ mixture) PSS (composition in mmol/l: NaCl 118.99, KCl 4.69, NaHCO_3_ 25, MgSO_4_.7H_2_O 1.17, KH_2_PO_4_ 1.18, CaCl_2_.2H_2_O 2.5, Na_2_EDTA 0.03, glucose 5.5), pH 7.4, at 37°C, a standardized computer-assisted normalization procedure was performed to set the pre-tension of the arteries. This defines the lumen diameter (l_100_) that the artery would have had in vivo when relaxed and under a transmural pressure of 100 mmHg. The arteries were then set to the lumen diameter l_1_=0.9×l_100_ (90% of the normalized inner diameter) when active force development was maximal. The change in wall tension (active wall tension) was calculated as measured force divided by the double segment length and expressed in mN/mm. Resting wall tension (which arises from the properties of the passive elements in the vascular wall) was also determined after the normalization procedure.

### Femoral artery reactivity

Before the start of measurements, the vessels were allowed to stabilize in PSS for 30 minutes. The experimental protocol consisted of the following steps: 45 min after normalization, PSS was changed to KPSS in which NaCl was exchanged for an equimolar concentration of KCl–in whole KPSS 125 mmol/l for 2 min–followed by wash-out with PSS (15 min). After noradrenaline (NA) addition (10 µmol/l, waiting to plateau) and wash-out (PSS, 20 min), pre-constriction was made by serotonin (Ser, 1 µmol/l, waiting to plateau). When the contraction of the femoral artery to Ser reached a steady state, increasing concentrations of the vasodilator acetylcholine (ACh, 0.001 to 10 µmol/l) were added in cumulative manner to perform endothelium-dependent concentration-response curves. When the concentration-relaxation curve was completed, the drugs were washed-out (PSS, 20 min) and the same experiment was repeated after 25-min pre-incubation with the nitric oxide synthase inhibitor N^G^-nitro-L-arginine methyl ester (L-NAME, 300 µmol/l) in the bath medium. After the following wash-out (PSS, 30 min), the nitric oxide donor sodium nitroprusside (SNP, 0.001 to 10 µmol/l) was added by cumulative manner to the pre-constricted arteries (Ser, 1 µmol/l). The same experimental protocol was repeated with PSS containing ascorbic acid (vitamin C, 1100 µmol/l) in the other segments of the same femoral artery. For technical reasons, determination of vascular reactivity without AsA started approximately at 8:00–9:30 a.m., while measurements with AsA started in the afternoon, approximately at 16:00–17:00 p.m.. In the meantime the arteries were kept in PSS and placed in the refrigerator at 4°C. Although it was shown that storage of the aorta in cold salt solution for 24 h did not alter the endothelium-dependent relaxation (Hansen & Nedergaard, 1999), we cannot rule out the effect of cold storage on endothelial function of the femoral arteries. Therefore we have not analyzed statistically the effect of AsA itself in control and stress conditions.

The NO-dependent component of endothelium-dependent relaxation was calculated as the difference between ACh-induced relaxation before and after acute L-NAME pre-treatment (Paulis *et al*., 2008) and expressed as area under the curve (AUC), in arbitrary units, based on the individual dose-response curves (Pruessner *et al*., 2003). In our preliminary experiments we ruled out tachyphylaxis (Hansen & Nedergaard, 1999) in two consecutive concentration-response curves for ACh in serotonin pre-contracted femoral arteries (data not shown). The extent of vasorelaxation was expressed in relative values as the percentage of Ser-induced contraction as well as in absolute values (mN/mm) to make sure that the changes measured were endothelium-dependent and not related to the extent of pre-constriction (Hansen & Nedergaard, 1999). Vasoconstrictions were determined as the maximal tension and they were expressed as active wall tension in mN/mm and also as effective active transmural pressure upon isometric activation at 90% of normalized inner diameter in kPa calculated on the basis of Laplace's equation (Mulvany & Halpern, 1977). Responses to each drug concentration were always allowed to stabilize before addition of a subsequent dose of the same drug or of another drug (Webb *et al*., 1987).

All chemicals used were purchased from Sigma-Aldrich (Germany), except noradrenaline hydrogenotartras (Zentiva, Czech Republic). All drugs were dissolved in distilled water and concentrations were expressed as final concentration in the myograph chamber.

### Oxidative stress markers

Conjugated dienes (CD) and concentrations of thiobarbituric acid-reactive substances (TBARS) were measured in the left ventricle and liver, as described previously (Hu *et al*., 1989).

### Nitric oxide synthase activity

NO synthase (NOS) activity was measured in the tissue homogenates of the aorta (200 mg/ml) by determination of [^3^H]-L-citrulline formation from [^3^H]-L-arginine (Amersham, UK), as described previously (Puzserova *et al*., 2006) and expressed as pmol/min/mg of proteins.

### Statistical analysis

Data are presented as group mean values ± standard error of the mean (SEM). Unpaired Student's t-test was used for comparison of means of two groups and two-way analysis of variance (ANOVA) for comparison of concentration-response curves. In case of significant result of two-way ANOVA, vertical contrast (pairwaise comparisons) with Bonferroni adjustment were performed. Homogeneity of variances and normality of distribution was tested by Levene's test and by Shapiro-Wilk's test, respectively. The significance level of all tests was set to 5% (α=0.05, *p<*0.05).

The concentration-response curves were fit by non-linear regression using four- parameter logistic equation with GraphPad Prism 5.0 software (San Diego, CA, USA).

## Results

### Basic cardiovascular parameters and oxidative stress markers

Basal blood pressure and heart rate of control and stressed rats before experiment were 107 ± 2 mmHg and 111 ± 1 mmHg, 349 ± 24 bpm and 378 ± 17 bpm, respectively. Chronic crowding failed to alter BP and HR *vs.* the control group (Table 1). At the end of the experiment, no differences in relative left ventricle weight and normalized internal diameter of the femoral artery were observed (Table 1). Similarly, there was no significant difference in the resting femoral artery wall tension of stressed rats *vs.* control rats (Table 1). Additionally, there were no significant differences in TBARS and CD concentrations of stressed rats *vs.* controls in either tissue investigated (Table 1). Chronic crowding increased significantly NOS activity in the aorta *vs.* control by about 76% (*p<*0.01; Table 1).

**Table 1:** Effect of chronic social stress on basic cardiovascular parameters and oxidative stress markers of Wistar-Kyoto rats.

	Control	Stress
Final BP (mmHg)	111 ± 3	112 ± 2
Final heart rate (bpm)	385 ± 18	352 ± 13
LVM/TL (mg/mm)	14.04 ± 0.48	13.24 ± 0.35
ND (μm) AsA-free	768.6 ± 12.4	794.6 ± 23.6
ND (μm) with AsA	780.3 ± 14.5	790.1 ± 14.8
WT (mN/mm) AsA-free	0.91 ± 0.07	1.03 ± 0.05
WT (mN/mm) with AsA	0.93 ± 0.13	1.02 ± 0.05
TBARS – LV (nmol/g)	8.25 ± 0.48	9.32 ± 0.47
CD – LV (nmol/g)	1054.44 ± 26.12	1065.19 ± 28.53
TBARS – liver (nmol/g)	15.00 ± 0.56	15.32 ± 0.79
CD – liver (nmol/g)	1585.56 ± 37.53	1605.56 ± 32.28
NOS – aorta (pmol/min/mg)	2.70 ± 0.24	4.76 ± 0.70**

Values represent mean ± SEM of 5–10 rats. Abbreviations: BP – blood pressure, LVM/TL – left ventricular mass-to-tibia length, ND – normalized inner diameter of the femoral artery, AsA – ascorbic acid, WT- resting wall tension of the femoral artery, TBARS – thiobarbituric acid-reactive substances, LV – left heart ventricle, CD – conjugated dienes, NOS – nitric oxide synthase activity

** *p<*0.01 as compared to control rats.

### Vascular responsiveness to vasoconstrictors

Both noradrenaline (10 µmol/l) and serotonin (1 µmol/l) induced contractile responses in the endothelium-intact femoral arteries. NA-induced responses were biphasic: a transient contraction (early response, phasic contraction) returned nearly to baseline and was followed by sustained contraction (delayed response, tonic contraction). Chronic crowding stress had no significant effect on the contractile responses induced by NA and serotonin (Table 2) in either AsA-containing or AsA-free PSS.

**Table 2:** Effect of chronic social stress on vascular constrictions induced by noradrenaline and serotonin of the femoral artery of Wistar-Kyoto rats.

	Control		Stress	
		
	(mN/mm)	(kPa)	(mN/mm)	(kPa)
Absence of AsA				
NA – phasic	1.07 ± 0.17	3.10 ± 0.51	1.06 ± 0.24	2.98 ± 0.70
NA – tonic	1.13 ± 0.19	3.25 ± 0.54	2.03 ± 0.84	5.80 ± 2.50
Maximal NA	1.32 ± 0.19	3.80 ± 0.54	2.14 ± 0.79	6.08 ± 2.38
Ser – before L-NAME	7.41 ± 0.15	21.43 ± 0.45	7.97 ± 0.15	22.34 ± 0.85
Ser – after L-NAME	8.94 ± 0.31^†††^	25.92 ± 1.12^††^	9.68 ± 0.30^†††^	27.17 ± 1.37^††^
Presence of AsA				
NA – phasic	0.75 ± 0.10	2.14 ± 0.27	0.84 ± 0.20	2.34 ± 0.51
NA – tonic	0.84 ± 0.07	2.39 ± 0.17	0.85 ± 0.14	2.36 ± 0.36
Maximal NA	0.85 ± 0.08	2.42 ± 0.18	0.90 ± 0.18	2.51 ± 0.47
Ser – before L-NAME	6.71 ± 0.47	19.18 ± 1.68	7.24 ± 0.35	20.36 ± 0.89
Ser – after L-NAME	8.09 ± 0.50	23.11 ± 1.86	8.72 ± 0.43^†^	24.57 ± 1.47^†^

Values represent mean ± SEM of 5–7 rats. Abbreviations: AsA – ascorbic acid (1100 μmol/l), NA – noradrenaline (10 µmol/l), L-NAME – N^G^-nitro-L-arginine methyl ester (300 μmol/l, 25 min), Ser – serotonin (1 µmol/l)

††† *p<*0.001

†† *p<*0.01

† *p<*0.05 as compared to control or stressed rats without L-NAME.

In the arteries pre-treated with the NOS inhibitor, the response to Ser was augmented as compared to responses before L-NAME administration (Table 2). However no changes were observed in stressed rats as compared to control rats.

### Endothelium-dependent and -independent vasorelaxation in absence of AsA

**Figure 1:**
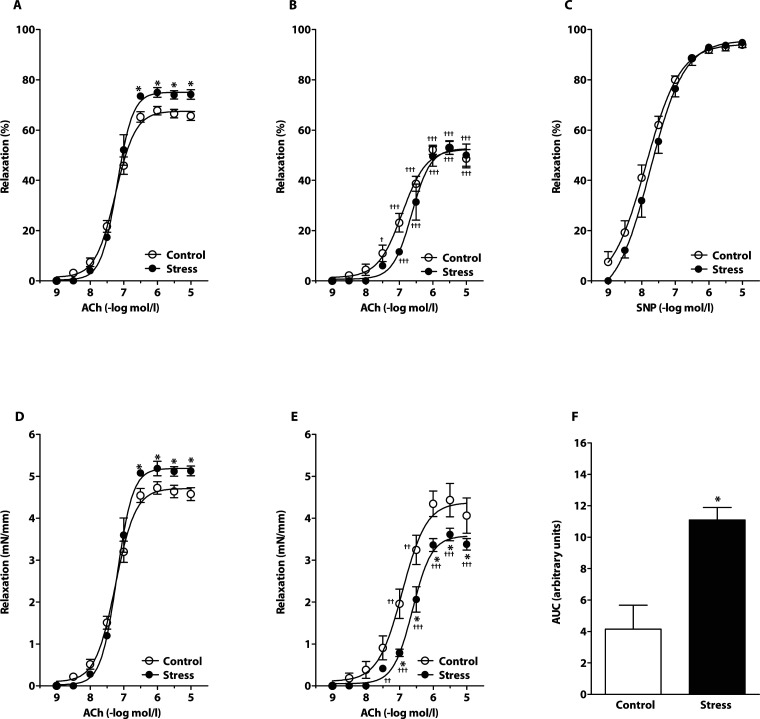
Effect of chronic social stress on acetylcholine (ACh)-induced relaxations in absence of ascorbic acid. Endothelium-dependent relaxations before (A,D) and after (B,E) incubation with the nitric oxide (NO) synthase inhibitor N^G^-nitro-L-arginine methyl ester (L-NAME); Sodium nitroprusside (SNP) – induced endothelium-independent relaxation (C); NO-dependent component of absolute ACh-induced relaxations (F). Values represent mean ± SEM of 5–7 rats. Abbreviations: AUC-area under the curve; *p<0.05, compared to respective value in control rats; ^†††^ *p<*0.001, ^††^ *p<*0.01, ^†^ *p<*0.05, compared to respective value in control or stressed rats without L-NAME.

Chronic crowding increased responses to ACh when expressed both as relative values to pre-constriction (Figure 1A) and absolute tension (Figure 1D). Stress also increased the maximal relative (76.24 ± 1.33% *vs.* 68.88 ± 1.68%, *p<*0.05, n=5–7) and absolute (5.27 ± 0.12 mN/mm *vs.* 4.80 ± 0.15 mN/mm, *p<*0.05, n=5–7) relaxation to ACh determined from the individual dose-response curves. Maximal relaxations were observed at ACh concentrations from 0.3 µmol/l to 10 µmol/l. Blockade of nitric oxide synthesis by L-NAME significantly reduced endothelium-dependent relaxations in both groups investigated (Figure 1B, 1E), however a significantly greater effect was seen in the arteries from stressed rats compared to controls (Figure 1E). The NO-dependent component of ACh-induced vasorelaxation (calculated from absolute relaxations) was significantly greater in stressed rats (Figure 1F).

Cumulative addition of sodium nitroprusside (SNP) produced similar relaxation responses in the femoral artery from stressed and control rats (Figure 1C). Maximal relaxations to SNP were also comparable in stressed and control rats (n.s.; 94.78 ± 1.96% vs. 93.94 ± 0.98% as expressed in relative values; and 5.93 ± 0.34 mN/mm vs. 6.28 ± 0.33 mN/mm as expressed in absolute values, n=5–7).

### Endothelium-dependent and -independent vasorelaxation in presence of AsA

**Figure 2:**
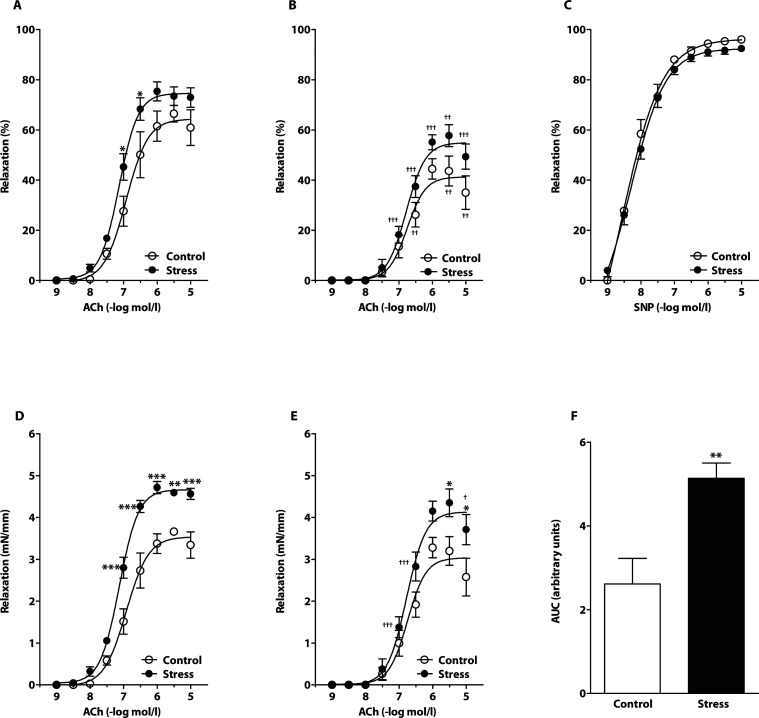
Effect of chronic social stress on acetylcholine (ACh)-induced relaxations in presence of ascorbic acid. Endothelium-dependent relaxations before (A,D) and after (B,E) incubation with the nitric oxide (NO) synthase inhibitor N^G^-nitro-L-arginine methyl ester (L-NAME); Sodium nitroprusside (SNP) – induced endothelium-independent relaxation (C); NO-dependent component of absolute ACh-induced relaxations (F). Values represent mean ± SEM of 5–7 rats. Abbreviations: AUC-area under the curve; ***p<0.001, **p<0.01, *p<0.05, compared to respective value in control rats; ^†††^ *p<*0.001, ^††^ *p<*0.01, ^†^ *p<*0.05, compared to respective value in control or stressed rats without L-NAME.

In the presence of AsA, chronic crowding increased responses to ACh when expressed both as relative values to pre-constriction (Figure 2A) and absolute tension (Figure 2D), similarly as observed in the absence of AsA. Blockade of nitric oxide synthesis by L-NAME significantly reduced endothelium-dependent relaxations in both groups investigated (Figure 2B, 2E). However, relaxations to ACh in the control group were unaffected after NOS blockade, when expressed in absolute tension (Figure 2E). Additionally, a significantly higher L-NAME-resistant component of vasorelaxation was seen in the arteries from stressed rats compared to control animals (Figure 2E). Stress elevated significantly the NO-dependent component of ACh-induced vasorelaxation (Figure 2F). SNP-induced relaxation responses were similar in rings from stressed and control rats (Figure 2C).

## Discussion

This study investigated the effect of chronic social stress on the cardiovascular system, oxidative load and vasorelaxation in normotensive WKY rats. Additionally, the effect of ascorbic acid on vasorelaxation and its NO-dependent component were investigated. The results showed that chronic stress had no effect on blood pressure, heart rate, relative left ventricular mass and diameter of the femoral artery. Similarly, chronic crowding failed to change endothelium-independent vasorelaxation and vasoconstriction as well as oxidative status in the left ventricle and liver. Interestingly, chronic stress caused a significant increase in endothelium-dependent ACh-induced vasorelaxation and its NO-dependent component in the femoral artery. This was associated with elevation of aortic NO synthesis in stress. Stress elevated NO-dependent vasorelaxation in stressed WKY rats in both the absence and presence of ascorbic acid.

Evidence that NO production may be considerably modified in stress and during adaptation to diverse stressors has entailed the hypothesis that NO plays an important role in stress and adaptive responses of the organism as a stress-limiting molecule (Malyshev & Manukhina, 1998; Cordellini *et al*., 2006). Since pharmacological reduction of NO production results in hypertension (Paulis *et al*., 2008; Torok, 2008), it may be assumed that the development of hypertension in chronically stressed rats could be associated with NO deficiency and/or endothelial dysfunction. However, the findings from experimental studies have not been consistent regarding blood pressure and endothelial function during chronic stress exposure. Similarly to our studies, several authors have failed to observe changes in blood pressure during chronic psychosocial stress in normotensive rats (Harrap *et al*., 1984; Henry *et al*., 1993). Moreover, Yamori *et al*. (1969) reported that chronic stress-loadings (immobilization; combined visual, auditory and electric stimuli; cold exposure) augmented hypertension and aggravated hypertensive lesions in spontaneously hypertensive rats, while the same stressors induced only a slight and transient elevation of blood pressure in normotensive Wistar rats. However, social stress produced by using large communal and complex population cages, resulted in stress-induced hypertension also in normotensive strains (Webb *et al*., 1987; Henry *et al*., 1993). In humans, an elevation of blood pressure was observed in prisoners when they were transferred from single occupancy cells to multiple occupancy dormitories, supporting the crowding theory (D'Atri *et al*., 1981).

As mentioned above, crowding is a relatively mild stressor, associated with increased interindividual interactions and reduced physical activity. Nevertheless, crowding affected plasma corticosterone levels and vascular function in rats depending on the cardiovascular phenotype. We showed that offspring of normotensive Wistar dams were able to adapt to this stressor via modification of vascular function. However, offspring of spontaneously hypertensive (SHR) mothers, either with borderline or fully developed hypertension, were not able of effective adaptation. Disturbed adaptation in borderline hypertensive and SHR rats was associated with significant increase of plasma corticosterone (Bernatova *et al*., 2007b) and with elevation of blood pressure (Bernatova *et al*., 2007a).

Regarding normotensive rats, similar crowding-induced changes were observed in our previous studies in Wistar rats (Bernatova *et al*., 2007a) and WKY (Puzserova *et al*., 2006) as in this study. Using the same stress protocol, we observed that chronic crowding increased ACh-induced relaxation of the phenylephrine pre-constricted femoral and the first branches of the superior mesenteric arteries of WKY rats (Puzserova *et al*., 2006; Bernatova *et al*., 2007c). Additionally, increased NO synthase activity and nitrate/nitrite levels were observed in the aorta (Bernatova *et al*., 2007c).

However in our previous experiments, in contrast to this study, vascular reactivity was investigated in modified physiological salt solution including ascorbic acid, a well-known antioxidant. Antioxidants are frequently used during investigation of vascular function *in vitro* to avoid degradation of some drugs, for example catecholamines (Paulis *et al*., 2008; Bernatova *et al*., 2009; Neves *et al*., 2009). Data from the literature indicate that the use of antioxidant additives might also be of importance because they modify the levels of vasoactive factors released by the endothelium (May, 2000; Bernatova *et al*., 2009). Reactive oxygen species (ROS) are known to interact with different endothelial vasoactive factors, mainly NO, and thus ROS can reduce NO bioavailability (Simonsen *et al*., 2009). Therefore the use of substances with antioxidant actions during investigation of vascular function *in vitro* might protect NO and other vasodilator factors from degradation and/or reaction with ROS and thus antioxidants may reduce endothelial dysfunction. However, in this study, we showed that crowding elevated responsiveness of the femoral artery to acetylcholine in both the presence and absence of ascorbic acid.

Since vasorelaxation, expressed as a percentage of pre-constriction, is inversely related to initial tension, we expressed relaxing responses also as absolute values (mN/mm) (Webb *et al*., 1987; Hansen & Nedergaard, 1999) to have certainty that changes measured are endothelium-dependent and not related to the extent of pre-constriction. Because acute L-NAME augmented the contractile responses to serotonin, the NO-dependent component of vasorelaxation was evaluated from absolute relaxations. In our study, in the absence of ascorbic acid, ACh-induced relaxations of the femoral artery from crowded rats were reduced after pre-incubation of vessels with L-NAME compared to controls (when they were expressed as absolute values), providing evidence for an elevation of the NO-dependent component of ACh-induced relaxation in stressed rats. Since NO-dependent relaxation is supposed to be eliminated by the given dose of L-NAME, the remaining ACh-induced relaxation (*i.e.* L-NAME-resistant component) should be related to other endothelium-derived relaxing factors, such as PGI_2_ and/or EDHFs.

Differences in the magnitude of overall vasorelaxation as well as in the NO-dependent component of relaxation in the presence and absence of AsA might be related to methodological aspects of the experiment, mainly to a delayed onset of experimentation with AsA (see Methods). Additionally, the reduced NO-dependent component of vasorelaxation in the presence of AsA (*vs.* absence of AsA) may result from the fact that in the given (relatively high) concentration AsA can act pro-oxidatively (Durackova, 2010) and therefore it can reduce NO bioavailability (May, 2000). Yet application of AsA had no impact on NO-dependent increase of vasorelaxation of stressed rats, suggesting that crowding did not produce significant oxidative stress in the femoral artery, similarly as observed in the LV and liver. However, since the basal oxidative status of hypertensive rats is considerably different from that of normotensive rats (Bernatova *et al*., 2009), the impact of social stress on oxidative load and thus on vascular function may vary from that in normotensive rats. Thus the use of AsA might reduce ROS level and endothelial dysfunction in hypertensive rats. On the other hand, the increase of the L-NAME-resistant component of relaxation in serotonin pre-constricted femoral arteries of stressed rats as compared to controls in the presence of AsA (this component was decreased in stressed rats in the absence of AsA) may reflect the fact that the use of different antioxidant additives in PSS may also be of importance and may modify the levels of vasoactive factors released by the endothelium.

It is known that NO is involved also in the regulation of vascular contractility. Using a variety of vasoconstrictors, impaired (Bernatova *et al*., 2007a), unchanged (Webb *et al*., 1987; Fuchs *et al*., 1998) or enhanced vasoconstrictions (Webb *et al*., 1987) were observed after chronic psychosocial and behavioral stress. The augmented contractile response to serotonin after acute L-NAME pre-treatment in this study was probably due to inhibition of nitric oxide production. However, our results suggest that chronic crowding did not modify NO production elicited by serotonin since the increment of serotonin-induced vasoconstriction was similar in control and stressed rats.

In our previous experiments, we found that phenylephrine and noradrenaline, which are usually used for pre-constriction of isolated arteries, caused only small and transient contraction of the femoral artery (Puzserova *et al*., 2006). That is why we used serotonin for pre-constriction of the arteries to evaluate vasorelaxation in this study. The selection of a different pre-contraction agent enabled us to investigate endothelial function under different *in vitro* methodological conditions, to make sure that the observed improvement of ACh-induced relaxation during chronic crowding stress did not originate from the given methodological approach. This is of importance because endothelium-dependent relaxation of the isolated arteries was indicated to be a function of the agonist used for pre-constriction of the artery (Li & Bukoski, 1993).

In addition, our data showed that alterations in NO-dependent relaxation exerted endothelial dependency because endothelium-independent relaxation to sodium nitroprusside in stressed rats was similar to that in controls. The data suggest that elevated endothelium-dependent, NO-mediated relaxation may be a protective mechanism which could counterbalance activation of the sympathetic nervous system during stress. On the other hand, persistent elevation of NO levels can result in generation of peroxynitrite, a potent oxidant and tissue-damaging agent (Leza *et al*., 1998). It is thus not clear whether NO would remain a protective molecule over a prolonged time-course of stress.

In conclusion, the results showed that the given model of chronic crowding stress did not elicit stress-related hypertension in normotensive rats. Stressed rats were able of effective adaptation associated with improvement of ACh-induced vasorelaxation and its NO-dependent component even if an ascorbic-acid-free working solution and a different way of pre-constriction were used, compared to our previous studies (Puzserova *et al*., 2006; Bernatova *et al*., 2007a, 2007c). Thus the results confirmed that elevation of NO production during chronic social stress is an important way of adaptation, which may prevent normotensive rats from the development of stress-induced hypertension via modulation of vascular function.
